# Estimation of Tool-Tissue Forces in Robot-Assisted Minimally Invasive Surgery Using Neural Networks

**DOI:** 10.3389/frobt.2019.00056

**Published:** 2019-07-16

**Authors:** Sajeeva Abeywardena, Qiaodi Yuan, Antonia Tzemanaki, Efi Psomopoulou, Leonidas Droukas, Chris Melhuish, Sanja Dogramadzi

**Affiliations:** Bristol Robotics Laboratory, University of the West of England, Bristol, United Kingdom

**Keywords:** sensor-less sensing, neural networks, minimally invasive surgery, haptic feedback, force estimation

## Abstract

A new algorithm is proposed to estimate the tool-tissue force interaction in robot-assisted minimally invasive surgery which does not require the use of external force sensing. The proposed method utilizes the current of the motors of the surgical instrument and neural network methods to estimate the force interaction. Offline and online testing is conducted to assess the feasibility of the developed algorithm. Results showed that the developed method has promise in allowing online estimation of tool-tissue force and could thus enable haptic feedback in robotic surgery to be provided.

## 1. Introduction

Robot-assisted minimally invasive surgery (RAMIS) has gained popularity in the last two decades through use of the da Vinci master-slave surgical system offering improved vision, precision and patient recovery time compared to traditional MIS (Lanfranco et al., 2004). However, certain shortcomings prevent RAMIS from fulfilling its maximum potential, including the lack of haptic feedback provided to the surgeon (Okamura, 2009). Attempts have been made to develop sensorised surgical instruments as a means to detect interaction forces during RAMIS and provide surgeons with haptic feedback. However, the size of force sensors and incision ports, the sterilization of tools at high temperature and the disposable nature of surgical tools have so far prevented integration of end-effector/tissue force sensing in RAMIS (Puangmali et al., 2008; Spiers et al., 2015).

Force estimation algorithms that do not require sensing hardware at the operating site include visual estimation of shaft deformation (Lindsey et al., 2009), modeling of surgical tool-tissue interaction (Okamura et al., 2004) and the use of motor current (Zhao and Nelson, 2015; Sang et al., 2017). Sang et al. (2017) modeled the dynamics of a da Vinci robot and, in conjunction with measured motor current, estimated the external force applied at the tip of the surgical tool; while Zhao and Nelson (2015) created a 3 degrees-of-freedom (DOF) surgical grasper prototype with joint dynamics modeled as individual linear 2nd order systems to estimate external forces (up to 2 *N*). These methods require some form of modeling and simplification (e.g., neglecting friction) which can affect the estimation accuracy. Further, the complexity of these algorithms may not allow for suitable update rates required for haptic feedback, thus affecting the systems overall stability and transparency.

Neural networks have been widely utilized both in adaptive control and for model approximations (Huang et al., 2000, 2002), thus constituting a worthy area of exploration for force estimation in RAMIS. Li and Hannaford (2017) used a supervised learning technique, Gaussian Process Regression (GPR), to estimate tool-tissue interaction. However, the GPR technique cannot predict well when the target is out of range of the training data. Further, neural network techniques can be combined with other estimation techniques. For instance, Yu et al. (2018) proposed a cable tension based method to estimate external forces and utilized a back propagation (BP) network to estimate resistance parameters such as friction to aid in the force estimation. Aviles et al. (2015) and Aviles et al. (2017) combined vision based methods with recurrent neural networks to estimate tool-tissue interaction.

In this work, we propose a neural network method to estimate the tool-tissue force interaction during a grasp manoeuvre in RAMIS for future application in providing haptic feedback to surgeons. Its novelty resides in the fact that it considers a black box approach regarding the whole mechanism, thus rendering the analysis of the grippers mechanism unnecessary. The proposed method is based on utilization of the current of the gripper-actuating motors as well as a detailed examination of the various stages of a grasping motion. In contrast to existing algorithms in literature, it does not require external sensors or equipment such as vision systems, predicting sufficiently both small and large forces. This paper is organized as follows; section 2 discusses the experimental setup, validates the use of neural network for force estimation and classifies the stages of a grasp; section 3 presents the proposed algorithm; section 4 provides results of the algorithm in offline and online situations and section 5 summarizes the major findings and proposes future work.

## 2. Methodology

### 2.1. Experimental Setup

**Figure 1:**
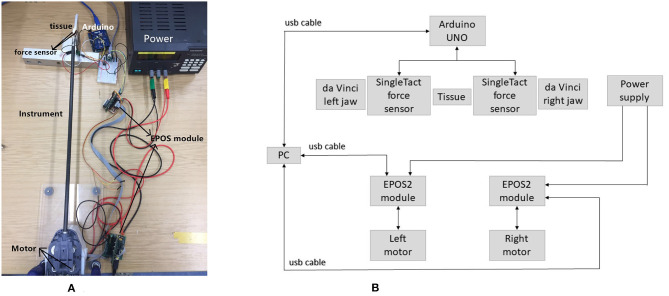
Experimental setup. **(A)** Da Vinci forceps were powered by two Maxon motors which interfaced with EPOS-2 modules. Applied forces were measured by two force sensors attached at the forceps jaws and interfaced with an Arduino. **(B)** Block diagram depicting the components and connections of the experimental setup.

The experimental setup, shown in Figure 1, utilized da Vinci forceps with four-degrees-of-freedom (dof) at the distal tip of the instrument (pitch, roll, yaw, grasp).

**Figure 2:**
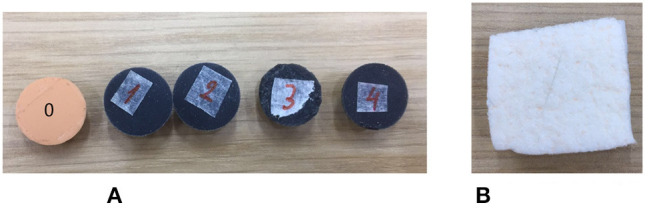
Samples used in the experiments to simulate tissue and tumors of various hardness. **(A)** 3D printed hemispherical objects were made from TangoPlus material. **(B)** Skin was simulated with a foam analog.

Grasping of tissue is an extremely common manoeuvre during RAMIS and is thus the focus of this work. As such, only yaw and grasp of the instrument tip were actuated in this investigation. Two Maxon brushed DC motors (3.89 mNm, 62:1 reduction) were attached to the da Vinci forceps via a custom-built housing. The motors interfaced with EPOS-2 modules (Maxonmotor, 2019) and were controlled in position mode. Two capacitive force sensors (SingleTact, 2019, 45 *N*, unidirectional) were attached to the jaws of the forceps and interfaced with an Arduino UNO (Arduino, 2019) to measure applied forces. The force measurement setup is detailed in Tzemanaki et al. (2018). Tissue and tumors were simulated by three-dimensional printed hemispheres (Stratasys 3D printer, TangoPlus material) and a foam tissue analog (Limbsandthings, 2019, dimensions: 1*m* × 0.42*m* × 15*mm*), shown in Figure 2, all with different hardness. Table 1 contains information regarding the various objects hardness that was measured with a Bareiss durometer in Shore A units (Bareiss, 2019).

**Table 1:** Hardness of 3D printed objects and artificial skin.

**Object #**	**Hardness (Shore A)**
0	99
1	65
2	52
3	41
4	27
Artificial skin	4

*Measured with a Bareiss durometer in Shore A units*.

**Figure 3:**
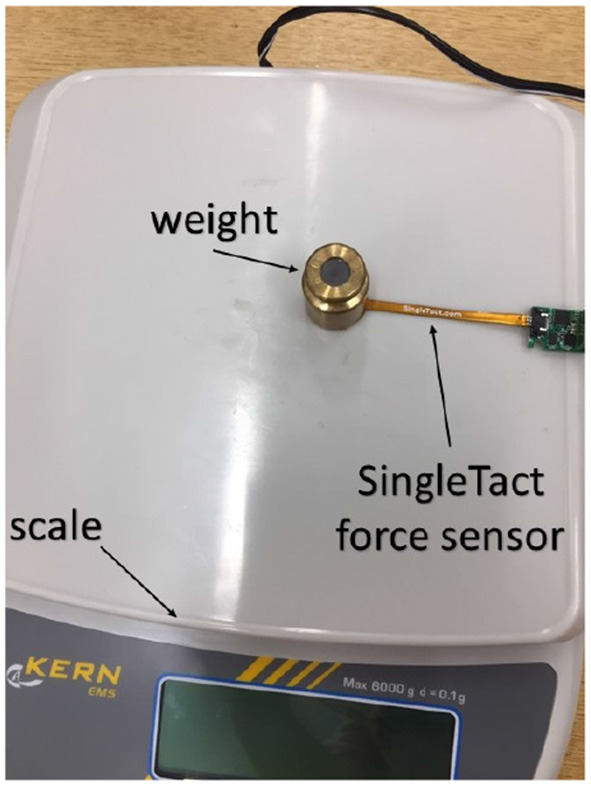
The force sensors were calibrated by applying known masses directly upon them, with a neural network mapping analog sensor reading to force (Newtons).

**Figure 4:**
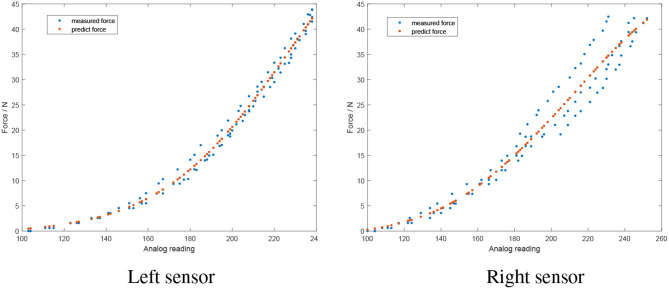
Results of the force sensor calibration (red dotted lines indicating the calibration curve). The sensors show similar performance with small differences due to individual calibration of each sensor.

The force sensors were calibrated by applying known masses directly on the sensors, as depicted in Figure 3. The corresponding analog output was read by the Arduino, logged and used as training data for the neural network. Each force sensor was calibrated individually, utilizing the same methodology for both sensors. Furthermore, experiments for each sensor were repeated three times to increase the accuracy of the calibration. A Multilayer Perceptron (MLP) neural network was utilized, which included one hidden layer with four nodes and a log-sigmoid transfer function. The input data to the network was the analog sensor reading and the output was the mapped force measured in Newtons (*N*). The networks properties were the same for both force sensors. The results of the calibration are shown in Figure 4. Since each sensor was calibrated individually, the calibration data may differ between the sensors but the overall curves are similar (Figure 4, red dotted lines).

### 2.2. Validation for Using a Neural Network

**Figure 5:**
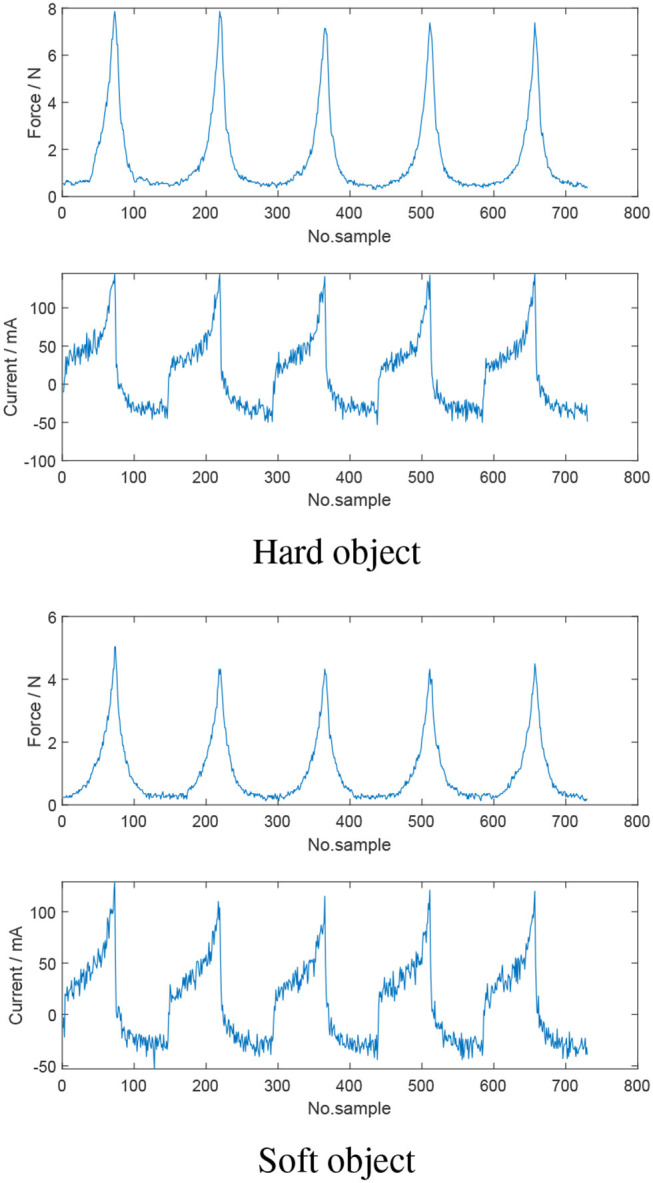
Comparison of the motor current and measured force when the jaws of the instrument are grasping hard and soft objects at the same commanded jaw angle. Harder objects resulted in larger applied jaw force and consequently higher motor current.

For a neural network methodology to be feasible, a relationship between the inputs and outputs of the network is required. As such, experiments were conducted using a hard and soft hemisphere. By controlling the motors position profile, the jaws of the instrument were commanded to grasp the hemispheres at the same jaw angle—with the force interaction between the jaws and hemispheres measured. From Figure 5 and Table 2, it is evident that interaction with the harder hemisphere resulted in a larger force. Further, it is evident that as motor current increases, the contact force increases. As such, it can be concluded that current and motor position can be used to estimate contact force and potentially classify tissue type.

**Table 2:** Maximum current for each grasp of hard and soft object, respectively.

**Grasp #**	**Hard object**	**Soft object**
1	145 *mA*	129 *mA*
2	144 *mA*	110 *mA*
3	141 *mA*	115 *mA*
4	143 *mA*	121 *mA*
5	145 *mA*	120 *mA*

### 2.3. Classification of Stages in a Grasp Manoeuvre

**Figure 6:**
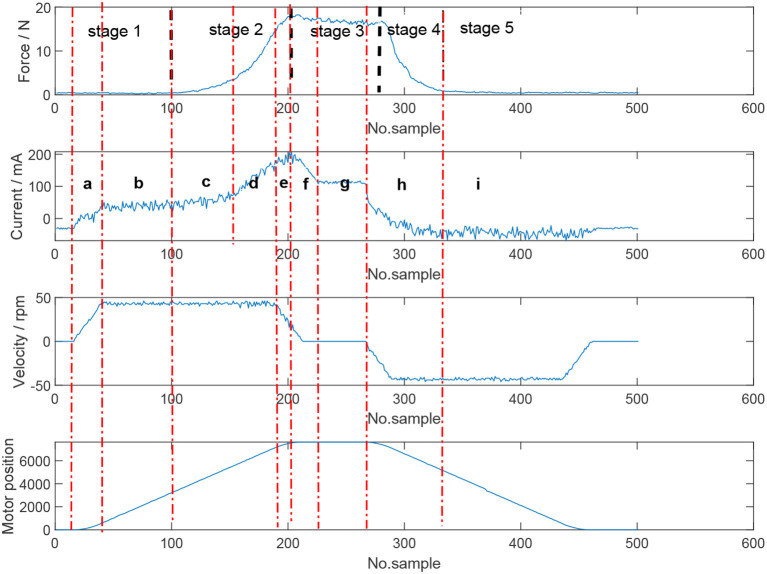
Stages of an entire object grasp and the characteristics of the force, position, velocity, and current profiles. Five stages can be identified based on the force profile: no grasp, beginning of contact, sustained squeeze/grasping, release of object, jaws and object no longer in contact.

The stages of a grasp and the characteristics in the force, position, velocity and current profiles are critical in determining a suitable neural network algorithm for force estimation. Consider Figure 6 which is an entire grasp of an object. Focussing on the force plot, five stages of a grasp manoeuvre can be identified. The first stage is when the instrument is not touching the object. During the second stage, the jaws begin to contact the object which results in the increase of interaction force. In the next stage, a sustained squeeze is applied on the object and the contact force remains fairly consistent. In the fourth stage, the jaws start to release the object and the force interaction decreases. The final stage indicates that the jaws and the object are no longer in contact. Consider the current figure in combination with force, position and velocity. Nine sections can be specified:

- First, the position, velocity and current of the motors increase;
- Once the velocity becomes constant, current follows a similar trend and the motors continue moving in the specified trajectory;
- When the jaw starts touching the object, the force interaction begins to increase and the current profile follows a similar trend;
- As the resistance between the jaw and object becomes greater, the slope of the force interaction and subsequently the current profile become steeper, as more current is required to drive the motors to the target position;
- With the motor approaching the target position, the velocity begins to reduce, and the force interaction and motor current reach maximum level;
- The motor has reached the target position and the velocity further decreases until it returns to 0. Correspondingly, the motor current also decreases and reaches a stable value at the same time that velocity becomes 0;
- The motor position is constant and a steady grasp is being applied on the object. Consequently, the force interaction and current remain fairly consistent;
- As the jaws are commanded to release the object; velocity, current and force all begin to decrease; and
- The jaws and object are no longer in contact. The force, velocity and current remain steady as the motor returns to its home position, with velocity returning to 0 when this position is reached.

### 2.4. Training Data

The quality of training data is critical for the neural network to be able to provide an accurate estimation. To develop a robust estimator, training data was obtained for objects with varying stiffness, i.e., soft sponge like to extremely rigid. Further, the data set consisted of grasps that applied a sustained force on an object (i.e., for around 20 s) and grasps that would squeeze an object then immediately release. The angle of the jaws was also varied such that the forces applied on the object had greater range.

## 3. Force Estimation Models

Due to the stages and signal characteristics of a grasp manoeuvre, the force estimation algorithm is separated into multiple models: the classifier, closing model, opening model and no-grasp model.

### 3.1. System Overview

**Figure 7:**
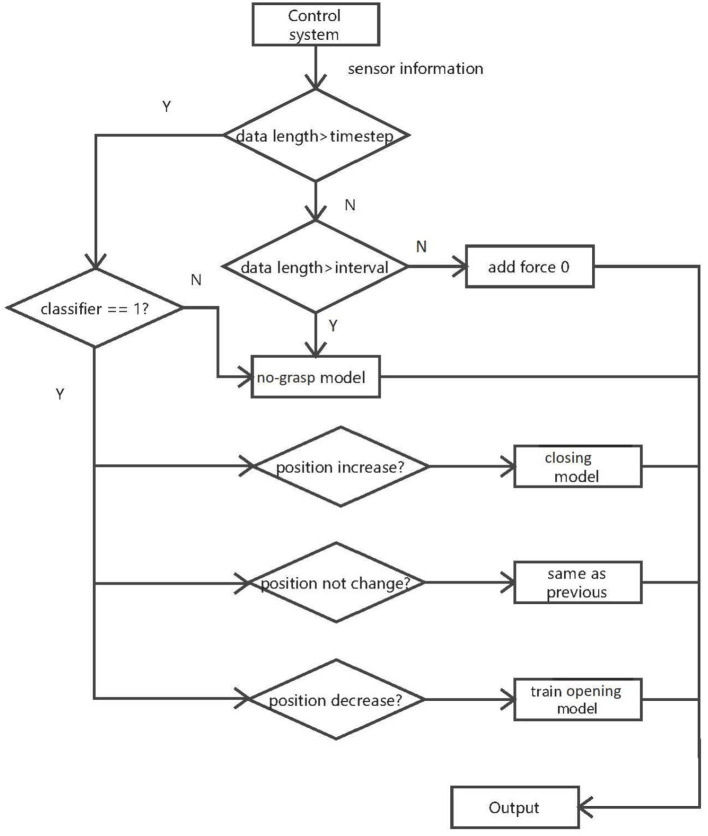
Structure of the estimation algorithm. The classifier detects if there is contact between the instrument jaws and object. The no-grasp model is applied when there is no contact between the jaws and object. The closing model is applied when the jaws are closing and grasping the object, and the opening model is applied when the jaws are opening and releasing the object.

Figure 7 represents the structure of the force estimation system. The control system box represents the data acquisition, i.e., sensor signals used for the algorithm; data length is the number of samples collected in the current state and timestep is used to determine if there is enough data to be sent to the classifier model. When the classifier returns a value of 1, the jaws are touching the object. The no-grasp model is used to map sensor information to contact force when the jaws are not in contact with the object, with interval setting a lower limit of data sent to this model. The angle of the motors are used to recognize the state of the instrument and which model should be used for force estimation.

The stages of the algorithm are:

1. When there is not enough data to be sent to the classifier, the force is assumed to be 0. If data length is bigger than interval but smaller than timestep, the processed input data is sent to the no-grasp model.
2. In stage 2, if data length is bigger than timestep, the classifier can be used. The classifier indicates if the jaws touch the object. According to the result of classifier, the data will be sent to different models. If the classifier says the jaws are not in contact with the object, the last interval data samples are sent to the no-grasp model. If the jaws are in contact with the object, then the data is sent to the next stage.
3. In stage 3, the instrument and the object are in contact. The current motor position in this state is compared with that in last state to identify the different states of the instrument (closing, constant or opening). If the current motor position is larger than the former, the closing model is applied to predict the contact force. If the motor position is constant, the output force is assumed to be constant. Otherwise, the opening model is used. This model is trained by information collected in the closing section of the instrument, including sensor values and predicted force. However, the opening model is trained only once the instrument is recognized to open. Other data collected when the jaws are releasing will be sent to trained opening model.

The classifier states that the jaws are either in contact with an object or not. When the jaws are in contact, three stages are apparent based on motor position. In these stages, the relationship between the inputs and the outputs are different as discussed previously in section 2.3. If the stages of a grasp could be predicted by one model based solely on the current profile, the force profile would not be symmetric because of the significant drop of current. However, the truth is that the measured force profile is symmetric. Velocity can be regarded as another potential input to build only one model for grasping but the velocity decreases before the instrument gets to the target position and can not help alleviate this issue. Based on the evidence, it is not ideal to build just one model to encapsulate one grasp. Therefore, separation is necessary. Regarding the sustained applied force section during grasping, i.e., Figure 6 (section f in Current plot), although there is a drop of current and velocity, motor position and force during this period do not change too much. Therefore, it is convincing that the determinant feature of this part is motor position. Hence, when in this state, the prediction system assumes the force is the same as the previous sample.

### 3.2. Classifier

The classifier model is used to determine when the jaws and object are in contact. This model was built to exclude the influence of tissue size, i.e., for two objects with the same properties but different size, to generate the same amount of force the smaller object will require a larger motor displacement. As such, the relative motor position is defined, i.e.,

(1) $$\begin{array}{r} \theta_{r}=|\theta_{c}|-|\theta_{s}| \end{array}$$

where θ_*r*_ represents the current relative motor position; θ_*c*_ is the current motor position and θ_*s*_ is the motor position the first time the classifier recognizes the contact of the jaws and object.

According to Figure 6, it is clear that when the jaws are closing, the slope of the current profile is larger than a near zero value. This corresponds to the contact of jaws and objects. When the current returns to a stable profile, the whole grasp move has finished. In order to detect the tendency, past information should be used. As such, the classifier relies on time-series data, with a long-short term memory (LSTM) network used. The LSTM network had 2 hidden layers each with 20 hidden units and used 40 samples. The LSTM layer was followed by a dropout layer to avoid any overfitting problems and a fully connected layer. A classification layer and softmax layer were used to conduct the classification tasks. The inputs to the LSTM network were motor current, position and velocity with the output being a flag that identifies the grasping status. Training data covered a range of tissue types to make a robust classifier network. The training data had the critical points of the grasp force manually set. Accuracy of the classifier was found to be 90%.

### 3.3. Closing Model

**Figure 8:**
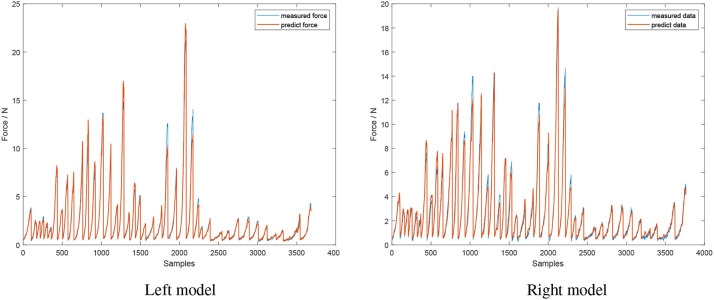
Performance of the closing model. Inputs of model's neural network are absolute value of current, velocity, and relative jaws position. Model's output is contact force (red line), compared to the actual measured force (blue line).

This model is used to describe when the jaws of the instrument are getting in contact with the object and in the process of squeezing. A recurrent neural network (RNNs) is dependent on previous information and is therefore unable to predict a sudden increase or drop in force from past information. As such, a feedforward neural network—the Levenberg-Marquardt backpropagation (LMb) method—was chosen to train the closing model. The network had 4 hidden layers each with 20 nodes and used a log-sigmoid transfer function. The inputs to this model were absolute value of current, velocity and the relative position of the left and right jaws, whilst the output was the contact force. The performance of the network is illustrated in Figure 8, with the mean square error of the left and right jaws being 0.20±0.44 *N* and 0.28 ± 0.46 *N*, respectively.

### 3.4. Opening Model

The opening model is trained using information from the closing model, as the distinguishing factor for force prediction is the relative jaw position. As such, this network is trained in an online manner and training time is critical. As such, a simple network is required. The relationship between input and output is simple in this case, so a complicated network could cause an over-fitting problem. Thus, a feedforward network with one hidden layer and 5 neurons was used.

### 3.5. No-Grasp Model

The no-grasp model is used when the jaws are not in contact with tissues. A feedforward network with the same network properties as the closing model was used. The inputs to the model are corresponding jaw position and motor current, with the output being the contact force.

## 4. Results

The developed algorithm is tested in two manners; first, in an offline manner to test if the prediction system is feasible, and then to see if the algorithm can estimate forces accurately in an online manner, such that it could have potential to be used in a force feedback system in RAMIS.

### 4.1. Offline Force Estimation

**Figure 9:**
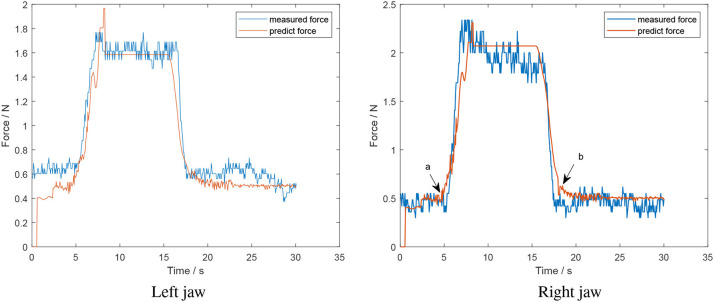
Offline test of a sustained squeeze of a hard object (estimated force from algorithm in red; measured force in blue).

In the off-line force estimation, the algorithm is run in a serial manner. The algorithm was tested for grasping manoeuvres that applied a sustained squeeze and a continuous squeeze and release motion. Figure 9 shows the result of the algorithm for a hard object with a sustained squeeze applied.

The predicted force follows the trend of the measured force; however, there is a discrepancy between the predicted model and the no-grasp model for the left jaw of 0.2 *N*. This region corresponds to when the jaws and object are not in contact with one another. This is not that consequential to the actual grasp force applied by the instrument, estimation of which is the primary goal of this work. Further, there is small sudden drop near the peak of the left jaw during the increasing section. At this point, the current of the motor suddenly decreased to zero and caused the drop in predicted force. A similar phenomenon is exhibited for the right jaw. Further, consider points *a* and *b* in the right jaw plot of Figure 9, which are the points at which the classifier identifies that the jaws and object have made and lost contact, respectively. The small increase is because the relative positions of the contact/lose contact are different, which affects the relative position for the decreasing section and causes the rise in force. The mean error for the left and right jaw for this situation was 0.12 ± 0.11 *N* and 0.13 ± 0.12 *N*, respectively.

**Figure 10:**
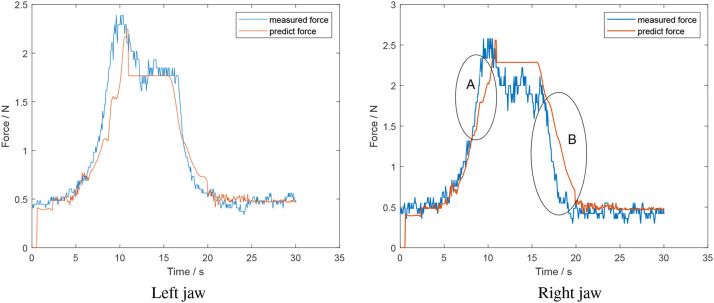
Offline test of a sustained squeeze of a soft object-foam tissue analog (estimated force from algorithm in red; measured force in blue).

Figure 10 is the performance of the system using a foam tissue analog. The force profile of the left jaw follows the trend of the measured force profile. However, at the end of the opening model section, a sudden decrease appears. This is the error of the corresponding classifier. In this case, it recognized the moment when jaws and objects lost contact before it actually occurred. According to Figure 10, in the opening model section, it seems that prediction force in right jaw decreases slower than the measured force (B). This is because the opening model depends on the closing model where the estimation rises slower than measured force (A). The mean error for the left and right jaw for this situation was 0.12 ± 0.16 *N* and 0.19 ± 0.18 *N*, respectively.

**Figure 11:**
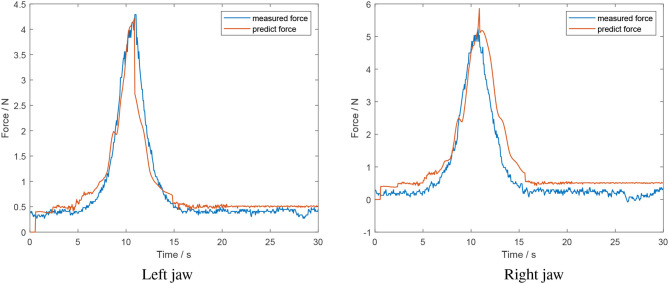
Offline test of a grasp and release of a soft object (estimated force from algorithm in red; measured force in blue).

Figure 11 is the result of a squeeze and release grasp applied on a soft object. It is evident that there is a spike and lag in the opening model section for the right jaw. This is due to a large relative position for the right jaw, with the model still believing it is in the closing model. As such, there is a spike and delay introduced in the decreasing grasp force estimation. The mean error for the left and right jaw for this situation was 0.17 ± 0.2 *N* and 0.39 ± 0.28 *N*, respectively.

In conclusion, the force estimation algorithm shows promise in fitting off-line data regardless of tissue properties; however, there is scope for improvement.

### 4.2. Online Force Estimation

The online system consists of two parallel routines; one related to hardware (control, data acquisition) and the other for online estimation of the contact force. The two routines were run in parallel and communicated via TCP/IP. The client was used for the control and data acquisition of the hardware and the server was dedicated to force estimation.

**Figure 12:**
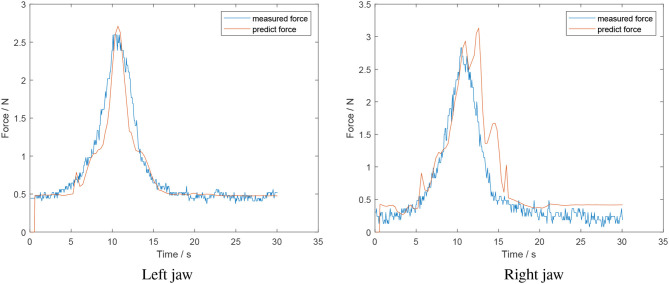
Online test of a grasp and release of a soft object-foam tissue analog (estimated force from algorithm in red; measured force in blue).

The result of an online test with the artificial skin is shown in Figure 12. It is evident that the force estimation for the decreasing section of the right jaw fluctuates. This can be attributed to the data available for training the opening model being limited. As such, the network overfits the available data.

In conclusion, the developed algorithm can predict the grasping force in an online manner. However, it is not robust and the execution time of the algorithm requires improvement such that sufficient data is available for the models to predict accurately.

## 5. Conclusion

This paper proposed a new algorithm using neural networks to estimate the grasping force of a surgical instrument for potential use in restoring haptic feedback in RAMIS. The results showed that the algorithm can estimate the tool-tissue interaction force during the grasp and has potential to be used in an online manner. This method can predict both large and relatively small forces which was an issue in the algorithm developed by Zhao and Nelson (2015). Moreover, the problem that GPR cannot predict the force out of training data's range can be solved using this algorithm. The main advantage of this algorithm is that it treats the whole mechanism as a black box so it is unnecessary to analyse the mechanism of the grippers. In addition, this method does not need to consider tissue properties compared to vision-based methods. Nevertheless, there exists scope to improve on the developed algorithm in the future. Namely, the execution time of the code should be improved for online estimation, uncertainty should be added into the models and potentially knowledge about the properties of the mechanism at hand (i.e., friction, tension). Moreover, since this work aimed to be a preliminary investigation to assess the feasibility of using motor current to estimate tool-tissue force interaction, a fixed position for the tool's wrist was assumed. Future experimentation will take into consideration grasping with different jaw configurations.

## Data Availability

The datasets generated for this study are available on request to the corresponding author.

## Author Contributions

SA and QY contributed conception of design, data acquisition analysis and interpretation, drafting/revising work. AT contributed conception of design, data acquisition analysis and interpretation. EP and LD drafting/revising work for important intellectual contribution. CM and SD provide approval for publication content and agree to be accountable for all aspects of the work.

### Conflict of Interest Statement

The authors declare that the research was conducted in the absence of any commercial or financial relationships that could be construed as a potential conflict of interest.
